# Using Existing Drugs as Leads for Broad Spectrum Anthelmintics Targeting Protein Kinases

**DOI:** 10.1371/journal.ppat.1003149

**Published:** 2013-02-14

**Authors:** Christina M. Taylor, John Martin, Ramakrishna U. Rao, Kerrie Powell, Sahar Abubucker, Makedonka Mitreva

**Affiliations:** 1 The Genome Institute, Washington University School of Medicine, St. Louis, Missouri, United States of America; 2 Department of Internal Medicine, Infectious Diseases Division, Washington University School of Medicine, St. Louis, Missouri, United States of America; 3 SCYNEXIS, Inc., Research Triangle Park, North Carolina, United States of America; 4 Department of Genetics, Washington University School of Medicine, St. Louis, Missouri, United States of America; McGill University, Canada

## Abstract

As one of the largest protein families, protein kinases (PKs) regulate nearly all processes within the cell and are considered important drug targets. Much research has been conducted on inhibitors for PKs, leading to a wealth of compounds that target PKs that have potential to be lead anthelmintic drugs. Identifying compounds that have already been developed to treat neglected tropical diseases is an attractive way to obtain lead compounds inexpensively that can be developed into much needed drugs, especially for use in developing countries. In this study, PKs from nematodes, hosts, and DrugBank were identified and classified into kinase families and subfamilies. Nematode proteins were placed into orthologous groups that span the phylum Nematoda. A minimal kinome for the phylum Nematoda was identified, and properties of the minimal kinome were explored. Orthologous groups from the minimal kinome were prioritized for experimental testing based on RNAi phenotype of the *Caenorhabditis elegans* ortholog, transcript expression over the life-cycle and anatomic expression patterns. Compounds linked to targets in DrugBank belonging to the same kinase families and subfamilies in the minimal nematode kinome were extracted. Thirty-five compounds were tested in the non-parasitic *C. elegans* and active compounds progressed to testing against nematode species with different modes of parasitism, the blood-feeding *Haemonchus contortus* and the filarial *Brugia malayi*. Eighteen compounds showed efficacy in *C. elegans*, and six compounds also showed efficacy in at least one of the parasitic species. Hypotheses regarding the pathway the compounds may target and their molecular mechanism for activity are discussed.

## Author Summary

Parasitic nematode infection is a large global health and economic problem, infecting around 2 billion people and costing $100 billion in crops and livestock. People in developing countries often live on one dollar per day, so treatments cannot be expensive, therefore using pre-existing drugs as lead compounds provides an economical way to begin to develop affordable treatments. Protein kinases were chosen as the focus of this work due to the large number of pre-existing drugs that target them and their important role in regulating almost all activities in the cell. Herein we describe a set of protein kinases conserved in diverse nematode species and experimental screening results of pre-existing drugs that target these kinases. The compounds that show *in vitro* efficacy in both *C. elegans* and parasitic nematodes, *H. contortus* or *B. malayi* have potential to be optimized further. These compounds have potential to provide accessible treatment to people in developing countries, as well as improving the health of livestock and boosting food production globally.

## Introduction

Abnormal and unregulated phosphorylation in signaling pathways can lead to diseases, such as cancer, diabetes, immunodeficiency, inflammation, and neurological disorders [1], [2]. Phosphorylation and dephosphorylation of proteins carried out by kinases and phosphatases regulate almost every activity in the cell [3]. Protein kinases (PKs) account for 2% of eukaryotic genomes [4] and are considered viable drug targets because the catalysis mechanism and overall structure of PKs are conserved. Further, it is well established that small molecules can bind to their catalytic cleft [5]. Hence, many kinase inhibitors have been developed to treat various human diseases, including drugs such as imatinib, trastuzumab, and lapatinib [2]. Understanding PKs can enable a deeper understanding of how signaling pathways effect development, pathology and biochemistry of an organism and also lead to more efficacious drugs [6]. In fact, PKs are considered the second most important group of drug targets after G-protein coupled receptors and are the largest enzyme family [3]. Although toxicity has been a concern in some cases, many drugs that target PKs have been approved for treating various diseases, despite some lacking specificity [3].

Given the importance of PKs in drug development, bioinformatics approaches and classification metrics have been developed to gain a greater understanding of PKs and PK inhibitors. PKs can be split into two diverse groups, with one group consisting of “conventional” PKs (ePKs) and the other comprised of “atypical” PKs (aPKs). The ePKs are the largest group and can be subdivided into 8 families and multiple subclasses using a multi-level hidden Markov model library [7]. The library consists of the following ePK classifications: the AGC family, CAMKs, the CK1 family, the CMGC family, the RGC family, the STE family, the TK family, and the TKL family. Proteins that do not fit into any of these classes are classified as other. The four aPK classifications consist of Alpha, PIKK, PHDK, and RIO. The multi-level library approach outperforms both BLASTP- and a Pfam HMMmodel-based approach in retrieving kinases and classifying them on a family level [7].

The World Health Organization estimates that over 2 billion people are infected with parasitic worms [8]. Further, parasitic worms also infect livestock and crops, which has deleterious effects on food production and has a negative economic impact worldwide [8]. Nematodes are becoming resistant to currently available anthelminthics and pesticides, thereby creating an urgent need to develop new compounds to combat these parasites [9], [10]. Protein kinases in nematodes offer novel targets for new drugs that are desperately needed to fight parasitic nematode infections throughout the world. Targeting PKs in parasites that cause diseases with high mortality and morbidity, such as malaria, have recently generated much interest, as recent studies have indicated specific inhibition of the protozoan kinases can be achieved [11]. Like protozoan parasites, anthelmintic drug development for nematodes could also benefit from studying nematode kinases. Kinases are evolutionarily conserved in eukaryotes, and the nematode *Caenorhabditis elegans*, has kinase orthologs for over 80% of the human kinome [4]. Given the large amount of information already existing for human PKs and kinase inhibitors and the overlap of kinases between *Homo sapiens* and *C. elegans*, kinases in parasitic nematodes are attractive targets for finding lead anthelmintic compounds. This strategy of target repurposing has been explored for initiation and prosecution of neglected disease drug-discovery programs (e.g. [12]). Furthermore, there are several examples in the literature where drugs have been also repositioned (e.g. [13], [14]).

By combining a variety of bioinformatics and cheminformatics approaches, along with laboratory screening on *C. elegans* and parasitic nematodes, we were able to learn more about kinomes of several nematodes spanning the phylum Nematoda. We identified kinases that are putative good targets, and experimentally test compounds that have been shown to interact with homologs of these kinases. Some of the compounds are already being used in the clinic or are in experimental phases of development for treating other diseases, making it possible to reposition this drug for use as a lead compound. For others, we have demonstrated anthelmintic potential, and due to their specificity, we also provide insight into pathways within Nematoda that might be important for drug targeting. Comparison of targets in nematodes and mammals also reveal opportunities for developing increased selectivity for nematodes.

## Results

**Figure 1:**
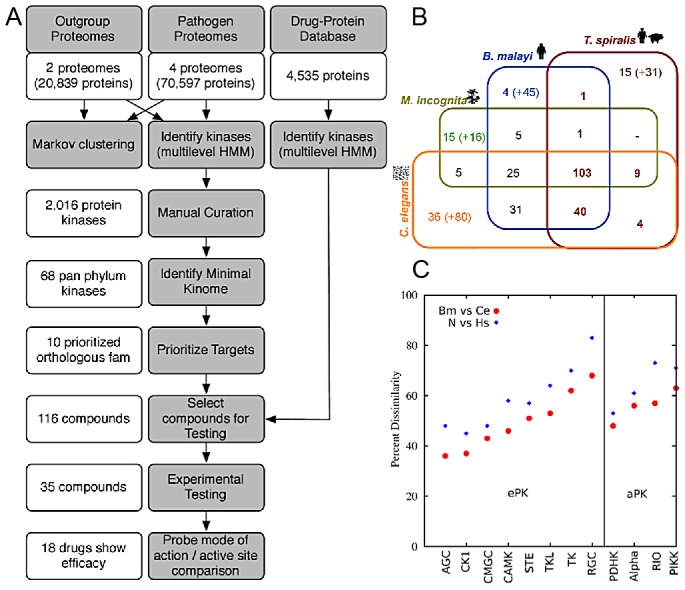
Methodology and pan-phylum kinome characteristics. A. Flow chart of methodology (gray) and compounds and target/compound elimination (white). B. Distribution of orthologous PK families among the nematodes spanning the phylum Nematoda. The orthologous groups were counted before the kinases underwent manual curation. The number in brackets are kinase genes not in groups C. Of the *C. elegans* kinases, orthologous groups were extracted that contain *C. elegans*, *B. malayi*, and *H. sapiens*. The *C. elegans* proteins were aligned to orthologous *B. malayi* proteins and each of these were aligned to orthologous *H. sapiens*. For each pairing, the identities were obtained through alistat [65]. Dissimilarity plot of nematode and human kinases using full-length sequence indicate different degrees of dissimilarity between species among kinase subclasses (e.g CAMK vs CMGC).

The methodology comprised a multi-step process, which commenced with the predicted proteomes of parasites and their hosts and resulted in prioritized targets and compounds (Figure 1A).

### Classification of Nematode and DrugBank Kinases

**Figure 2:**
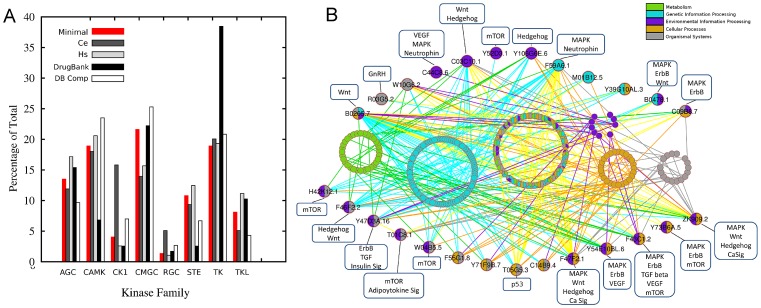
Minimal kinome analysis. A. Nematode minimal kinome compared to *C. elegans* (Ce), *H. sapiens* (Hs), DrugBank Targets, and drug target of each compound in DrugBank. Nematode minimal kinome compared to *Ce*, *Hs*, DrugBank targets, and drug target of each compound in DrugBank. B. Nearest neighbors from the minimal kinome clustered based on KEGG Pathways. The minimal kinome is shown as large circles and the nearest neighbors are small circles. The nodes are colored based on the KEGG pathways in which they participate.

PKs from each genome were identified (Figure 1B & Figure S1) and the 294 orthologous groups containing PKs were phylogenetically classified. The 133 PKs previously shown to be shared by 3 nematode species [15] decreased to only 103 (or 68 when only the manually curated kinases are considered) when the plant-parasitic Thylenchid, *Meloidogyne incognita* [16], and the zoonotic parasite, *Trichinella spiralis* [17] were included (Figure 1B). The number of kinases shared among the nematode species could be underestimated due to the draft nature of the parasitic nematode genomes. The 68 members of the pan-Phylum conserved kinome are referred to as the minimal kinome. The minimal kinome is dominated by kinases from the TK, CMGC, and CAMK groups (Figure 2A). For each nematode genome, the manually-curated kinases from the minimal kinome are listed in Tables S1, S2, S3, S4. The most prevalent groups in *C. elegans* include: CK1_sub1, CMGC_sub3, CMGC_sub2, RGC_sub1, and TK_sub2 (Kinomer with custom cutoffs) (Figure S2). The most prevalent groups in *B. malayi* include CMGC_sub2, CMGC_sub3, TK_sub2, CK1_sub1, AGC_sub4, and STE_sub1. The largest groups in *H. sapiens* include CMGC_sub2, CAMK_sub2, AGC_sub4, CMGC_sub3, TK_sub2, and AGC_sub4.

**Figure 3:**
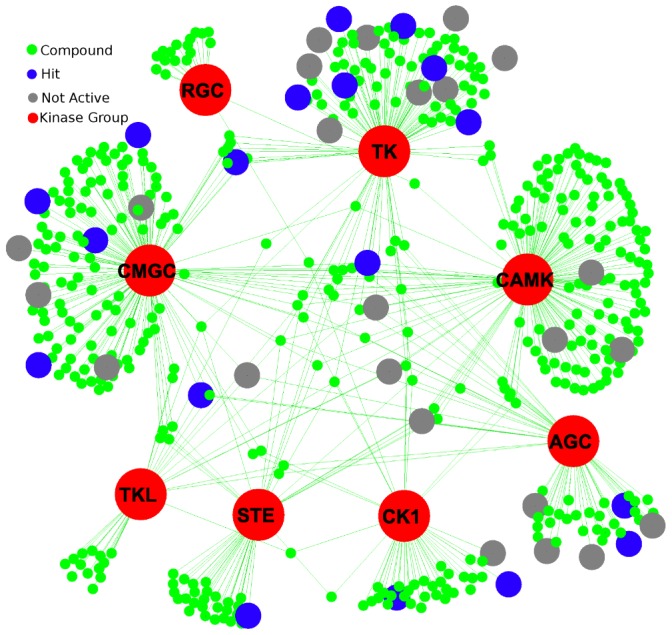
Drugs from DrugBank and their interaction with the different kinase groups. The kinase groups are shown in red, and the blue nodes indicate compounds that yielded a phenotype in *C. elegans*. The grey nodes were tested, but didn't yield a phenotype.

The kinases in DrugBank were also characterized in a similar manner as the nematode kinases. There are 519 compounds in DrugBank that target one or more of the 299 kinases using kinase models from Kinomer [7]. Interestingly, CAMK_sub1, CMGC_sub3, CMGC_sub2, and TK_sub2 all have the largest number of compounds that bind targets in that group (Figure S2). Some compounds are very specific for a particular kinase group, whereas others can bind to targets in multiple kinase groups (Figure 3).

Primary sequence similarity among nematode full-length PKs is higher than between nematodes and *H. sapiens* (Figure 1C), providing opportunities for specific targeting, despite having similar active sites. For example, when all isoforms are included, the 68 groups contain 153 proteins from *C. elegans*. Using cutoffs intended to identify kinases that were very different from humans, an amino acid-based similarity search among nematode and human kinases yielded 138 *C. elegans* kinases that were above the cutoffs, and the remaining 15 had weak homology to *H. sapiens*. Even within the conserved orthologous groups, kinases have substantially diverged in the host (Figure 1C) while still maintaining similar active sites necessary to carry out kinase function. Furthermore, for example, the dissimilarity level between nematode CAMK members and nematode/human CAMK members was much larger than between the nematode CMGM and nematode/human CMGM members (Figure 1C).

### Target and Compound Selection

The classification of kinases and compounds that target them resulted in 116 compounds to screen and many gene candidates as well. To reduce the number of compounds and potential targets, only orthologous groups that had a protein conserved across all four nematode species that span the Phylum were considered because of their potential for broad control. Using the sixty-eight orthologous PK groups found in the previous section dramatically reduced compound search space. Out of 38 total kinase subgroups, only 28 kinase subgroups had orthologs in all four nematode species. 22 orthologous groups, spanning 13 kinase subgroups, had an RNAi phenotype in *C. elegans*. Of the 22 orthologous groups, 14 (spanning 12 kinase subgroups) also have anatomical expression data in *C. elegans*. After considering RNAi phenotypes, nine orthologous groups were found which had expression in specific tissues known to be useful for drug targeting (i.e. pharynx [18], intestine [19], muscle [20] etc) and manifested a RNAi phenotype. These groups include: TK_sub2, AGC_sub4, CMGC_sub1, TKL_sub4, STE_sub1, CK1_sub2, TKL_sub2, and TKLK_sub5. Three other orthologous groups were identified because the groups had expression patterns conducive to being a drug target, but did not have an RNAi phenotype: CAMK_sub4, AGC_sub2, and AGC_sub4. The groups meeting these criteria, along with RNAi phenotypes, and stage and tissue expression, are shown in Table S5. RNAi phenotype and phenotype that resulted from the addition of compound could be different, depending on the region targeted by RNAi in multiple experiments. A similar phenotype obtained by the compound and the RNAi screen indicates that the compound has a mode of action that targets the same gene the RNAi targets; however, different phenotypes do not preclude a similar mode of action.

For our compound testing, we wanted to maximize the number of kinase groups tested with compounds, while also being cost effective. As a result, major kinase groups that appeared more than once in the top hit list were eliminated partially based on the number of compounds that target the subgroup: STE_sub4, TLK_sub2, and TLK_sub5. TLK_sub2 and STE_sub4 had only 3 and 5 compounds, respectively, associated with them in DrugBank. Based on the subgroup classification, 116 unique compounds that target kinases in eight kinase subgroups (Table S6) were investigated, 35 were prioritized based on cost and accessibility for experimental testing (Table S7). Specificity was not used to prioritize compounds.

### Compound Screening in *C. elegans*

**Figure 4:**
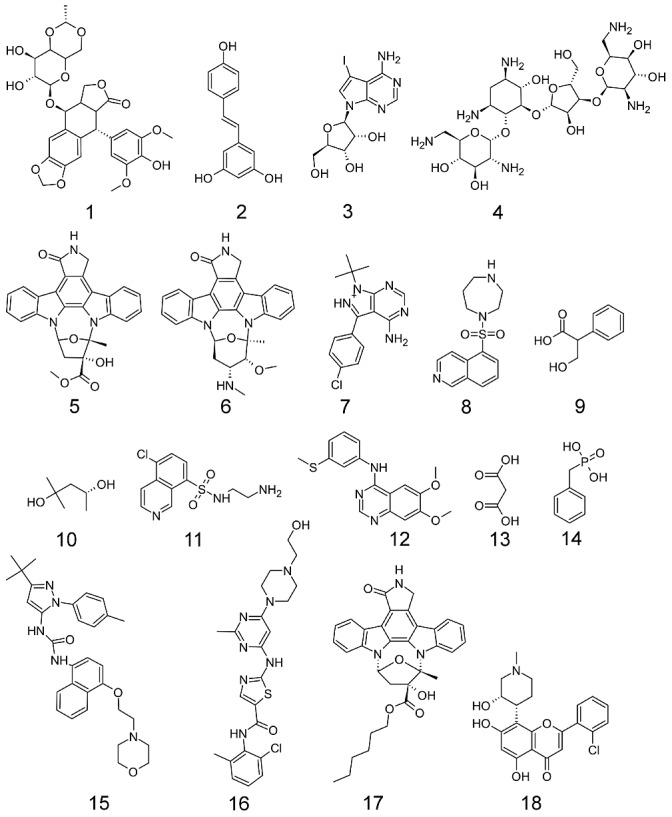
Structures of compounds that elicited a phenotype in *C. elegans.* **1** (DB00773) Etoposide, **2** (DB02709) Resveratrol, **3** (DB04604) 5-iodotubercidin, **4** (DB03615^s^) Neomycin, **5** (DB02152) K-252a, **6** (DB02010) staurosporine, **7** (DB03023) PP2, **8** (DB04707) HA1077, **9** (DB04080^s^) Tropic Acid, **10** (DB03684) 2-methyl pentanediol, **11** (DB03693) N-(2-Aminoethyl)-5-Chloroisoquinoline-8-Sulfonamide, **12** (DB02984) 4-[3-Methylsulfanylanilino]-6,7-Dimethoxyquinazoline, **13** (DB02175) malonic acid, **14** (DB02908^s^) Benzylphosphonic Acid, **15** (DB03044) BIRB 796, **16** (DB01254) Dasatinib, **17** (DB01933) KT-5720, **18** (DB03496) Flavopiridol.

**Figure 5:**
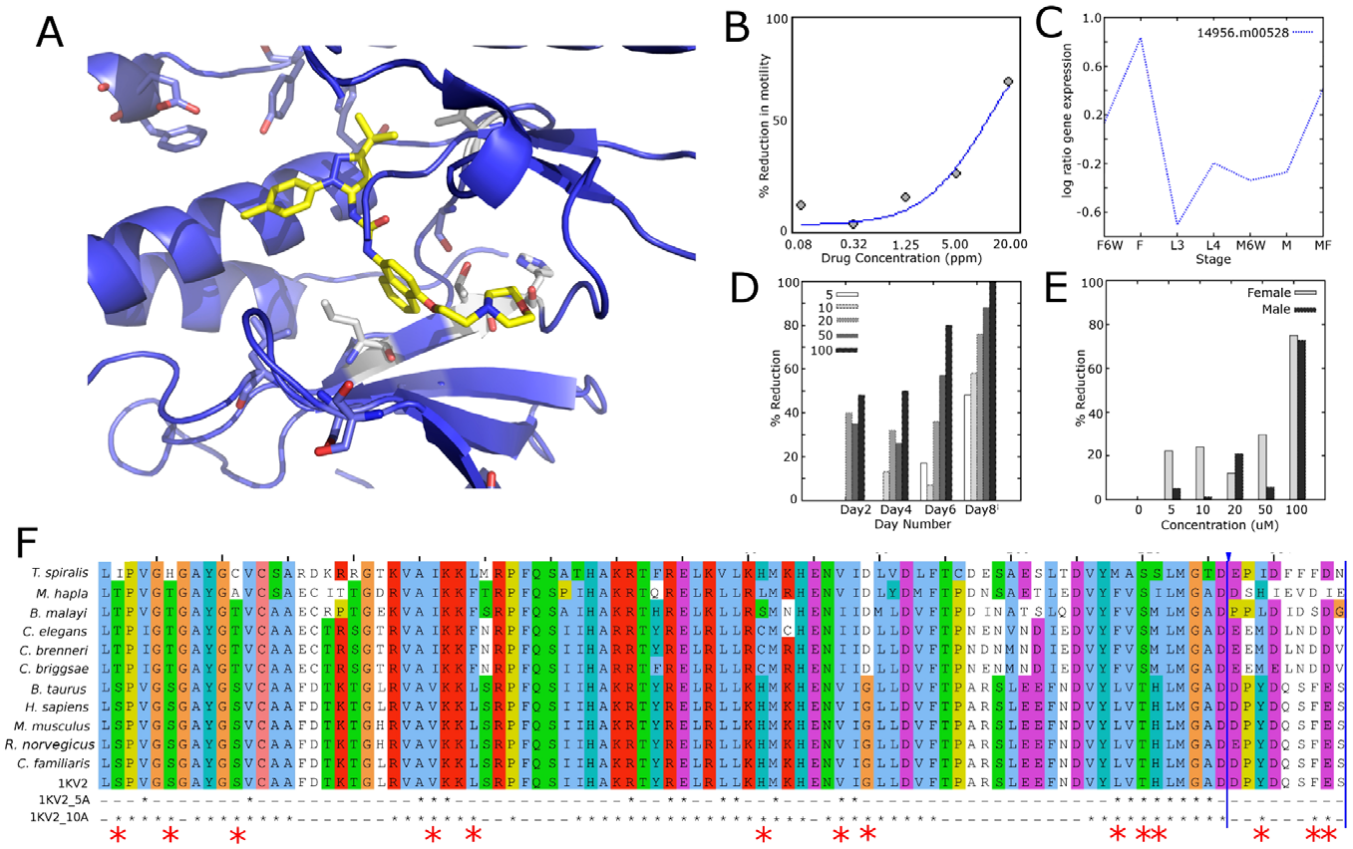
Alignment, structure, and experimental data for p38 MAP kinase (CMGC_sub1). (**A**). 1KV2 with **15** bound. Residues that differ between mammals and nematodes within 5 Å of the active site are colored in grey, and residues within 10 Å are shown in blue. The compound is colored yellow. (**B**). Dose-response curve of **15** in *C. elegans*. (**C**). Expression data from *B. malayi* microarray data [66] for p38 orthologs in *B. malayi*. (**D**). Percent reduction in microfilarial release in *B. malayi* cultured with **15** *in vitro*. (**E**). Percent reduction in viability in *B. malayi* female and male worms cultured with **15**. (**F**). 1KV2 alignment of Tsp_3128 (*T. spiralis*), Mh10g200708_Contig883_17801_19594 (*M. hapla*), 14956.m00538 (*B. malayi*), B0218.3 (*C. elegans*), CBN12041 (*C. brenneri*), CBG01555 (*C. briggsae*), NP001095644.1 (*B. taurus*), ENSP00000229795 (*H. sapiens*), ENSMUSP00000004990 (*M. musculus*), P70618.3 (*R. norvegicus*), NP_001003206.1 (*C. familaris*), 1KV2. Residues 5 and 10 Å from the compound in the active site are starred. Differences in the active site are represented by larger red star at the bottom.

The compound screening began with L1-stage worms, which developed into adult worms over the course of the 72-hour experiment. Expression of target *C. elegans* genes over these stages would be ideal to strongly indicate the target was present for the compounds to bind. Thirteen compounds exhibited an EC_50_<20 ppm and 12 yielded a detectable phenotype. In all, 18 compounds (Figure 4) yielded a detectable phenotype and/or generated an EC_50_ less than the maximum dose tested (Table 1). EC_50_ values were calculated based on the concentration at which 50% of the nematodes were not moving. Phenotype was not assessed by EC_50_. The *C. elegans* were exposed to five different concentrations of compound in duplicate, and the effect was subsequently independently confirmed by a separate experiment on a different set of worms. An example dose-response curve (for **15**) is shown in Figure 5B, and the rest are shown in Figure S3. Selected videos taken at 20 ppm are included as Supplementary Videos (Video S1, S2, S3, S4).

**Table 1:** Compound screening in *C. elegans*, *H. contortus, and B. malayi*.

				*C. elegans* a	*H. contortus*	*B. malayi*
Comp Num	DrugBank ID	Common Name	*C. elegans* (EC^50^ µM)	Pheno	(MIC90 µM)	*(MEC* µM)^b^
**1**	DB00773	Etoposide	>34.0	+	>20	
**2**	DB02709	Resveratrol	>87.6	+	>20	
**3**	DB04604	5-iodotubercidin	2.6	+	>20	
**4**	DB03615^c^	Neomycin	9.7	+	>20	
**5**	DB02152	K-252a	15.3	−	1.3	
**6**	DB02010	Staurosporine	0.7	−	5	
**7**	DB03023	PP2	46	+	>20	
**8**	DB04707^c^	HA1077	28.4	−	20	
**9**	DB04080^c^	Tropic acid	120.4	−	>20	
**10**	DB03684	2-Methyl-2,4-Pentanediol	123.5	+	>20	
**11**	DB03693	N-(2-Aminoethyl)-5-Chloroisoquinoline-8-Sulfonamide	>70.0	+	>20	>100
**12**	DB02984	4-[3-Methylsulfanylanilino]-6,7-Dimethoxyquinazoline	>61.1	+	>20	100
**13**	DB02175	Malonic Acid	>192.2	+	>20	
**14**	DB02908^c^	Benzylphosphonic acid	63.5	−	>20	
**15**	DB03044	BIRB 796	36.3	−	>20	10
**16**	DB01254	Dasatinib	22.3	−	>20	
**17**	DB01933	KT-5720	36.9	+	>20	
**18**	DB03496	Flavopiridol	48.3	+	>20	

a *C. elegans* Phenotype 72 hrs post treatment: jerky or slow movement, twitchy, and combination of these.;

b Minimum effective concentration where effect is seen in 50% of worms;

c Similar to DrugBank compound within a Tanimoto similarity score of 0.8.

### Compound Screening in Parasitic Nematodes *H. contortus* and *B. malayi*

The 18 compounds that yielded a detectable phenotype in *C. elegans* were also tested in an *H. contortus* larval development assay. Three compounds (**5**, **6**, **8**) had a MIC_90_ lower than the maximum dose tested (Table 1). The dose-response curves are shown in Figure S4, and the selected videos taken at 20 µM are shown in the Supplementary Videos (Video S5, S6, S7, S8).

Based on their target specificity in DrugBank, three compounds (**11**, **12**, **15**) with activity in *C. elegans* were chosen for *in vitro* testing in *B. malayi* for antifilarial activity. Results showed motility was affected by **15** at 100 µM concentration (by Day 4) (Figure 5D); the other compounds did not affect motility. In addition, **15** markedly reduced viability by Day 8 and 72–75% reduction in worm viability was observed in both female and male worms, respectively (Figure 5E). Compound **15** inhibited microfilaria (MF) production in a time and concentration-dependent manner. After 2 days in culture with **15**, female worms showed greater than 50% inhibition in MF release at high concentrations (100 µM). By Day 8, 10 µM appeared to be the minimum effective concentration that could inhibit MF release from female worms. Although there was no effect on motility and viability, a 50% reduction in MF release was observed by **12** at Day 6 (Figure S5).

## Discussion

Therapies are desperately needed to combat parasitic nematode infections, which plague over 2 billion people or 1/3 of the earth's population [21]. Since helminth infections are endemic in developing countries, we explored possible repositioning of existing drugs for use as lead compounds, as using a pre-existing drug lowers substantially the cost of drug development. The nematode kinome is a rich resource for exploring compounds that may show anthelmintic activity due to the wealth of existing information on PKs and PK inhibitors. The lead compounds found via repositioning could be modified to increase efficacy. Using a pan-phylum compilation of nematode proteomes, kinases were identified and classified into groups and subsequently into subgroups. Kinases in DrugBank were also identified and classified into groups/subgroups. Compounds were linked to targets via the subgroup classification, and disparate information regarding the kinase targets was combined to prioritize the targets (and associated compounds) for experimental testing. A total of 35 compounds were tested in *C. elegans*, and 18 exhibited a deleterious phenotype.

With our upfront prioritization and characterization, our study had a much higher hit rate with respect to *C. elegans* nematodes (51%), compared to a previous study where high-throughput screening of ∼14,000 compounds was done and resulted in 308 compounds yielding phenotypes (2.2% success rate [22]). Out of the ∼14,000 compounds 483 are likely to be kinase inhibitors in the entire library based on a cheminformatic search using a Tanimoto score of 0.8 (3.45%), and of the 308 displaying phenotype (3.24%) 10 were kinase inhibitors. Our hit rate was 18/35 (∼51%) compared to the HTS approach 10/483 (2.1%), resulting in a 25× enrichment. While the difference in the concentration used by the two studies (∼25 µM versus 20 ppm (∼25 to 60 µM, depending on the molecular weight of the compound)) could in part be responsible for the observed enrichment, our prioritization approach highly enriches for good potential candidates. Only two of our compounds tested overlapped with the high-throughput screening study [22]; **2** caused jerky and abnormal movements in *C. elegans*, and the other (DB01953) did not produce a phenotype. Furthermore, our strategy used species that span the phylum, increasing the possibility of identifying candidate targets for broad control.

At the root of our study was i) identification of the kinome within each of the nematode species, and ii) identification and characterization of a minimal kinome, or group of kinases that are conserved among species that span the phylum Nematoda. The 68 pan-phylum nematode PK orthologous groups spanned 9 different PK groups (TK, AGC, CMGC, TKL, STE, CAMK, CK1, RIO, and RGC) and 28 subgroups (Table S4) out of a possible 12 groups and 38 subgroups. These kinases were linked to kinases in DrugBank based on their subgroup classification. A representative of each of the ePK groups is present in the minimal kinome, but some groups were more highly represented in the minimal kinome than others (Figure 2A), with CMGC being the most highly represented (21.6%). The highest reduction in parasitic nematodes versus free-living was detected in the Receptor Guanylate Cyclases (RGC) that has been shown to be expanded in several metazoans, most dramatically in *Caenorhabditis* species [23]. Based on our analysis, there is a drastic reduction of this class in the parasitic nematodes compared to *C. elegans*, especially in the human parasites *T. spiralis* (2%) and *B. malayi* (1.7%) vs *C. elegans* (5.1%) (Figure S1). The nematode kinase groups had corresponding drug targets (by associations because drug targets were classified the same way) and drugs in DrugBank. DrugBank has nearly twice as many TK targets, most likely due to their prominence as cancer targets. However, DrugBank had nearly two fold more compounds that target CAMK proteins relative to the total number of compounds and targets, which is mainly caused by a large number of compounds targeting CAMK_sub1 (Figure S2).

Over 65% of the minimal kinome that successfully maps to the major KEGG pathways are involved in Environmental Information Processing including the MAPK, Wnt, mTOR, or ErbB signaling pathways (Figure 2B); however, only 34% of the minimal kinome can be mapped to the KEGG pathways. When the minimal kinome is mapped to KEGG pathways along with the proteins with which they interact, the vast majority are involved in genetic information processing or a combination of different pathways (Figure 2B). The entire *C. elegans* minimal kinome maps to all five major KEGG pathways; however, individual kinase groups often lack a specific major pathway. For instance, TK and RGC are the only groups that map to metabolic pathways and CAMK and TK do not map to any genetic information processing pathways (Figure S6).

The second phase of our study involved linking compounds in DrugBank to kinases in nematodes that were conserved across the phylum. This study did not bias the results toward kinase inhibitors, rather all compounds in DrugBank were considered if the target made the appropriate cutoff. Thus, some compounds are not typical kinase inhibitors, but had experimental evidence for binding to a kinase. These compounds were tested so as to not throw away potential lead compounds, even though they were not typical kinase inhibitors. The 18 compounds that yielded a phenotype in *C. elegans* provide excellent lead compounds that could be developed into anthelmintic drugs. Further, 6 of the 18 compounds also showed efficacy against at least one of two very different nematode species, the blood-feeding *H. contortus* and the filarial *B. malayi*. The difference in lifestyle between *C. elegans* and the parasitic nematodes is quite large, making the finding that these 6 compounds are more broadly applicable to the entire phylum. However, differences in the species could also lead to a large difference in compound potency across the species, as the species have different uptake mechanisms. Further, the screening on the various species included different life stages, which could also lead to differences in compound potency. Several of these compounds that were hits in *C. elegans* are already FDA approved drugs used as antimicrobials and/or cancer drugs. One example is **16** (Dasatinib), an approved small molecule that targets BCR/Abl in chronic myloid leukemia, which exhibited deleterious effects on worms yielding an EC_50_ of 22.3 µM. Flavopiridol, **18**, is an experimental treatment for cancer that also yielded an EC_50_ of 48.3 µM. Neomycin, **4**, is an antibacterial compound that yielded an EC_50_ of 9.7 µM. Etoposide, **1**, is an approved small molecule and has antitumor activity which caused jerky/abnormal movements in *C. elegans*. These compounds have already extensive toxicity data for humans associated with them (**16** [24], [25]; **18** [26]–[28]; **4** [29]; **1** [30]–[33]).

Detailed examination of the results, allowed formation of hypotheses regarding the pathways in which the targets are involved and the compounds might be affecting. It is not surprising that the pathways are hard to deconvolute based on looking at compound-protein interactions. Of 276,122 bioactive compounds, 35% were known to bind to multiple targets, and surprisingly, 25% of these bind to proteins in different gene families [34]. In cases such as TK_sub2, the targets are all very similar even though this is a particularly large group. Within TK_sub2, 13 compounds were tested, and 7 of them yielded an EC50 >20 ppm and/or a phenotype in *C. elegans*. Oftentimes, the compounds are able to target several different kinases within this group, making it particularly difficult to discern a precise targeted protein or pathway.

**Figure 6:**
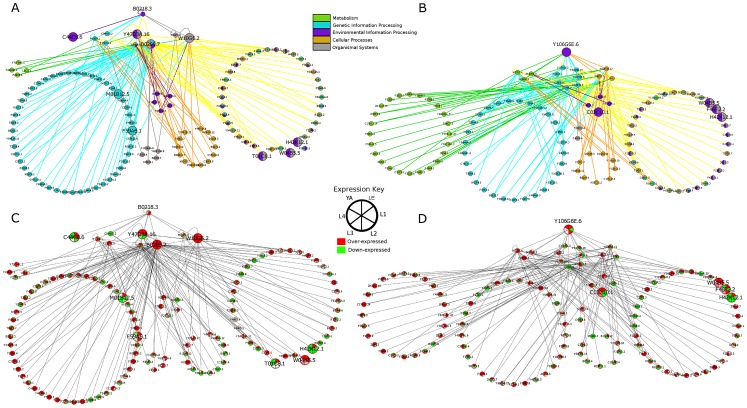
PPIs from MINT mapped using PPIs from *C. elegans* and *C. elegans* orthologs from *D. melanogaster* and *S. cerevisiae*. PPIs for A. B0218.3 and C. Y106G6E.6 color coded and grouped based on KEGG pathway assignment. PPIs for B. B0218.3 and D. Y106G6E.6 color coded by expression levels from RNAseq data. The proteins involved in pathways connected to B0218.3 are linked to several different pathways, including genetic information processing. MAPKs play important roles in signal transduction and are involved in cellular processes such as proliferation, differentiation, and cell survival in eukaryotes. B0218.3 is differentially over-expressed in the late embryo (LE) and L1–L3 stages (p<0.1) based on our analysis of *C. elegans* expression data [59].

For all the compounds, additional studies need to be done to completely confirm the compounds' mode of action. However, three subgroups of kinases, which had compounds that were likely to specifically target a particular enzyme, provide useful hypotheses into the compound's mode of action for further study. The first group is CMGC_sub1, which is a kinase group that contains predominately CDKs (cyclin dependent kinase) and MAPK (mitogen-activated protein kinase). Of the 14 *C. elegans* proteins in this group (cutoff 1e^−5^), 4 were conserved among *T. spiralis*, *B. malayi*, and *M. incognita*. These conserved *C. elegans* kinases are B0285.1 (cdc2 kinase), B0205.7 (casein kinase II), F43C1.2 (ERK5), and B0478.1 (JNK). From DrugBank, there are 14 drug targets that are classified in CMGC_sub1. Out of the eight compounds tested that target this group, two compounds, **12** and **15**, showed efficacy and specifically target p38 based on literature searching and DrugBank listings. In a previous study [35], **15** bound to several of the *H. sapiens* orthologs of *C. elegans* proteins in the minimal kinome: B0285.1 (1100 nM), F43C1.2 (2500 nM), B0478.1 (9100 nM and 7.3 nM). However, **15** binds to the *H. sapiens* ortholog of B0218.3 (NP_620581.1) at a much higher affinity (0.45 nM), increasing the likelihood that B0218.3 is the main target in our *C. elegans* screen. The proteins with which B0218.3 interacts include those involved in organismal systems and environmental information processing and are also differentially over-expressed during stages in which the compound screening assay was carried out (Figure 6A & C).

The p38 kinase, B0218.3, is only conserved among *C. elegans* and the vertebrate parasites (*B. malayi* and *T. spiralis*); it was not present in the minimal kinome because a p38 ortholog is not found in the plant parasite, *M. incognita*. Inhibitors of p38 have shown promise in fighting other parasite infections [36]–[39]. Our results suggest that p38 may be a good target for other filarial parasitic nematodes species as well. Structural analysis can be done, as X-ray crystal structures exist of **12** and **15** bound to p38. Although most residues in the active site are conserved between mammals and mouse, some nearby residues differ (Figure 5A & C and Figure S5A & S5B), creating opportunities for development of more specific drugs. Further, **12** and **15** were tested in *B. malayi* adult worms and were found to have an effect. The expression results [40] also support the sex-dependent effect on worm motility in *B. malayi*. **12** caused a reduction in worm viability in female worms, but caused no reduction in male worms (Figure S5C–E). **15** caused a reduction in worm viability in female worms at a much lower concentration than in male worms (Figure 5C–E). Compound **15** is currently in phase III clinical trials for rheumatoid arthritis and Crohn's disease [41].

The second group for which the mode of action could be hypothesized is CK1_sub2, providing an excellent basis for further experimental testing. Involved in key regulatory processes [42], casein kinase 1 has been shown to be an important drug target for various parasitic species [43]–[46] (Figure S7). Out of the 3 compounds tested that target this group, 2 compounds, **11** and **3**, showed efficacy. Compound **11** specifically targets casein kinase 1 from CK1_sub2. Compound **11** did not yield an EC_50_ value, but appeared to have jerky movement. Compound **3** binds casein kinase 1, as well as ser/thr protein kinase haspin and MAPK3, yielding an EC_50_ value of 2.6 µM and resulting in worms that move slowly and are smaller and less developed. X-ray crystal structures are available: **3** bound to CK1 in *Saccharomyces pombe* and **11** bound to *H. sapiens* CK1-gamma (Figure S8). Comparing their sequence to *C. elegans* proteins that were classified into CK1_sub2, the sequence of the X-ray crystal structure most closely resembles Y106G6E.6, making it likely that the compounds bind to this protein and its other casein kinase I gamma nematode orthologs (Figure S7). Further, Y106G6E.6 is also differentially over-expressed in late embryo, L2, and young adult stages (Figure 6D), which include stages during which the screening experiment was done. The results from *B. malayi* further point to the compound targeting its CK1 alpha orthologs. Compound **11** did not pass the threshold for causing a 50% reduction in motility, but came very close in males with 41.6% reduction in viability in males at 100 µM concentration. (Figure S9). Given the effect was sex-associated, it is likely that **11** is also targeting the CK1 alpha orthologs (Figure S7).

The third group that had a compound that specifically targeted one enzyme is AGC_sub2. One hit from this group, **17**, selectively inhibits Protein Kinase A (PKA). Compound **17** is not in DrugBank, but was chosen for testing because of its similarity to DB01933 and the inability to obtain DB01933. DB01933 targets AGC_sub4, but **17** is a known inhibitor of PKA, which is in AGC_sub2. Not only did **17** yield an EC_50_ when added to *C. elegans*, the worms also displayed jerky, slowed, and twitching movements on the test plate, in addition to being smaller. Interestingly, siRNA was used to knockdown a splice variant of PKA, causing paralysis in the *C. elegans* adult stage [47]. Compound **17** is a derivative of **5** and **6** which target other kinases non-specifically. Compound **8** (similar to DB04707 – hydrofasudil) also targets PKA, but also inhibits Rho-associated protein kinase (ROCK), as well as several other targets not listed in DrugBank (protein kinase G, NADPH oxidase, myosin light chain kinase, etc). Compound **8** is already used to treat cardiovascular disease [48] and has the potential to be a promising therapy to manage severe malaria [49]. PKA has been suggested as a good drug target in the filarial nematode, *Onchocerca volvulus* [50], and in the protozoan, *P. falciparum* [51].

In this study, we have classified kinases in several different nematode species, *C. elegans*, *B. malayi*, *T. spiralis*, and *M. incognita*, which span several phylogenetic clades and lifestyles (free-living and parasitic) in the phylum Nematoda. The proteins were placed in orthologous groups, and 68 orthologous groups had proteins in each of the nematode species, indicating those proteins are conserved and therefore important for nematode survival. Drug targets in DrugBank were also classified, and compounds that bind to DrugBank targets were matched with promising nematode proteins via kinase classification. Several PK inhibitors, which are currently being used in the clinic or in experimental phases of development for other diseases, were tested and shown to have efficacy in *C. elegans*, *H. contortus* and*/*or *B. malayi*. For the compounds that show efficacy, we also made hypotheses about their mode of action via bioinformatic and structural analysis and provide some insight into how to improve specificity for the nematode versus mammalian protein in several cases.

## Materials and Methods

A flowchart of the methodology is shown in Figure 1.

### Ethics Statement

All animals were handled in accordance with guidelines defined by the Animal Welfare Act (A3381-01), Association for Assessment and Accreditation of Laboratory Care International (AAAALAC), PHS Policy for the Humane Care and Use of Laboratory Animals, the Guide for the Care and Use of Laboratory Animals, and the Division of Comparative Medicine, Washington University School of Medicine. All animal work was approved under WUSM Institutional Animal Care and Use Protocol 20120025.

### Classification of Nematode and DrugBank Kinases

Kinase domain models were downloaded from the Kinomer website (http://www.compbio.dundee.ac.uk/kinomer/allPK.hmm) and were used to screen a collection of gene sets from the organisms *Brugia malayi*, *Caenorhabditis elegans*, *Drosophila melanogaster*, *Homo sapiens*, *Meloidogyne incognita*, *Saccharomyces cerevisiae*, and *Trichinella spiralis*. Custom score thresholds per kinase group were taken from Miranda-Saavedra [7] and then adjusted until an hmmpfam search (HMMER v2.3.2) came as close as possible to identifying all known *C.elegans* kinases using the Kinomer allPK.hmm profile database. Those same cutoffs were then applied to the gene sets of the remaining 6 organisms, identifying sets of putative kinases in each case. These putative kinases were categorized into the 8 conventional kinase groups (ePK) and the 4 atypical kinase groups (aPK) by merit of which model they were found to hit in the Kinomer profile database (allPK.hmm).

We then manually curated the sets of putative kinases by screening them against Pfam using hmmpfam (as a part of an interproscan run (interproscan software v4.5, interpro db release 2.2)) and making sure that no clear contradictions were found. Any cases where a putatively identified kinase was found to have a clearly non-kinase Pfam domain hit, were removed from the final set of identifications. The custom cutoffs used per kinase group were as follows: TK, 5.5e-03; CAMK, 9.6e-07; CK1, 1.1e-02; CMGC, 6.7e-03; AGC, 1.1e-14; STE, 3.4e-03; RGC, 4.8e-05; TKL, 8.7e-03; PDHK, 4.7e-160; PIKK, 1.4e-06; Alpha, 8.5e-66; RIO, 7.5e-10.

The same methodology used to classify the nematode kinases was also used to classify targets in DrugBank. DrugBank v2.5 was used to screen against the kinase domain models using an E-value cutoff of 0.1. If the target in DrugBank and a nematode kinase were classified into the same kinase subgroup by the HMM, the nematode protein by association was hypothesized to interact with one of the drugs in DrugBank known to bind a particular target.

### Prioritization of Kinases to Test

OrthoMCL [52] was used to group the 6 proteomes (*B. malayi*, *D. melanogaster*, *M. incognita*, *T. spiralis*, *S. cerevisiae*, *C. elegans*) into orthologous groups using a default inflation factor of 1.5 (all othologous groups are available at Nematode.net [53]). To determine the best kinase groups which have compounds that target them, the kinase groups were evaluated as to whether they exhibited an RNAi phenotype and if there was tissue expression data in *C. elegans*. RNAi phenotypes for *C. elegans* (http://www.wormbase.org/#012-3-6, WS220; downloaded on April 19, 2011) were grouped based on Kumar et al. [54]. The complete list of RNAi phenotypes, sorted by bin, are available as Table S2 in Taylor, et al. [55]. Tissue expression for *C. elegans* was obtained from Wormmart [56] on April 23, 2010. GO associations of the all helminth proteins were made by running InterProScan [57] (release 4.5). EST [58], tissue localization, and RNAseq data [59] were also used to evaluate potential groups for compound testing.

### Protein-protein Interactions and Pathway Information

Protein-protein interactions from MINT [60] were found for *C. elegans*, *S. cerevisiae*, and *D. melanogaster*. Using the protein orthologous groups obtained from the OrthoMCL clustering, the protein-protein interactions in *C. elegans* were expanded using orthologous protein-protein interactions in *S. cerevisiae* and *D. melanogaster*. Cytoscape [61] was used to analyze the networks, and the multicolor node plugin [62] was used to analyze kinase classification and expression values.

### Compound Screening in *Caenorhabditis elegans*

Compounds for experiments were obtained from the following sources (compounds obtained are shown in parenthesis): Enzo LifeSciences (1,2,4,7,8,14,17), Ryan Chemicals (9, 10, 11, 12, 13, 15), and LC (5,6) and TRC (3,16,18). Some of the compounds were not found commercially, so these compounds were substituted with available compounds with the highest similarity assessed via Tanimoto score of 0.8 or greater, which was calculated using OpenBabel [63]. Compound screening commenced with L1 stage worms and ran for 72 hrs at which point the *C. elegans* were around adult stage, so expression of the conserved *C. elegans* genes over the course of the experiment is required to maximize the effect of the compound on the organism. RNAseq data was taken into account when selecting compounds to test, but was not used to limit the compounds tested. For example, genes expressed during the early embryo stage may not be functional until the L1 stage. Thirty-five compounds were chosen for testing in *C. elegans* based on compound availability and cost at various vendors.

Compounds formulated in 100% DMSO were tested in microtiter plates containing 50 µl nematode growth media, 1% *E. coli* and 20 L1 *C. elegans*. The efficacy of a compound was determined based on the motility of the larvae as compared to average motility of control wells containing DMSO only at 48 hours post treatment. Larval movement was manually assessed at 72 hours post treatment to determine if there were altered movements or morphological changes not detected by the imaging system. *C. elegans* was exposed to the five different concentrations (0.08–20 ppm; ∼25 to 60 µM, depending on the molecular weight of the compound) and two replicates, and the effect was subsequently confirmed by an independent test.

### Compound Screening in *Haemonchus contortus*

Compounds were screened in *H. contortus* after formulation in 100% DMSO. The testing was performed in microtiter plates containing 50 µl nematode media, fecal slurry and 20 L1 *H. contortus*. *H. contortus* was exposed to five different concentrations (0.08–20 µM). The efficacy of a compound was determined based on the motility of the larvae as compared to average motility of control wells containing DMSO only. A MIC_90_ value was calculated by determining the lowest dose at which there was a 90% reduction in motility as compared to the control wells. Larval movement was manually assessed at 72 hours post treatment to determine if there were altered movements or morphological changes not detected by the camera.

### Compound Screening in *Brugia malayi*

*B. malayi* adult male and female worms were obtained from the peritoneal cavities of male gerbils infected with third-stage larvae at 120 days post-infection with third stage larvae. Worms were washed 3 times with RPMI-1640 to eliminate host cell contamination and then washed 3–4 times with RPMI containing 200 U/ml streptomycin, 100 µg/ml penicillin, and 0.25 µg/ml of amphotericin-B (Sigma).

#### Effect of 3 kinase inhibitors on parasite motility and viability

Effect of inhibitors were studied by incubating two adult female and male worms (4) in 2 ml of either culture medium (CM) alone or in CM with added compounds dissolved in solvent DMSO in 24 well culture plates. Final concentration of DMSO in CM was less than 1%. Control cultures with worms were set up with different dilutions of solvent in medium only, and additional worm cultures included only 2 ml of culture medium. Compounds were added to the culture medium at a final concentration of 5 µM, 10 µM, 20 µM, 50 µM and 100 µM. The culture medium was replaced with fresh medium with compounds on alternate days and all cultures were terminated on day 8. The worm cultures with or without compounds were carried out in duplicate and results were expressed as means of the replicate experiments [64].

#### Parasite motility

Parasite motility and death was assessed visually by microscopy and the observations were scored as 0, immotile or dead; 1, slightly active; 2, active and motile; 3, moderately active and motile; and 4, highly active and motile (equal to the activity and motility of worms cultured in culture medium without added compounds).

#### MTT reduction assay

Parasite (adult worm) viability was assessed quantitatively by the MTT reduction assay [64]. Briefly, single worms were placed in 0.5 ml of PBS containing 0.5 mg/ml of MTT and incubated at 37°C for 1 hr. At the end of the incubation time, worms were removed and carefully transferred into a separate well of microtiter plate containing 200 µl of DMSO and kept at room temperature for 1 hr. The absorbance of the resulting colored product was then determined at 490 nm wavelength using a microplate reader. Dead worms that had been heat killed and doxycyline (10 µg/ml) killed were used as negative controls for MTT reduction experiments. The minimum concentration of compound that caused >50% reduction in MTT test was considered as significant.

### Microfilaria Counts

Adult female worms incubated in CM released MF *in vitro* under the culture conditions tested. MF released into the CM counted in duplicate 20 µl aliquots on 2, 4, 6 and 8 days post-culture. Results were expressed as the percent reduction in MF release relative to results obtained with control worms cultured in CM without added compounds.

## Supporting Information

Figure S1 **Distribution of PKs per major families in distinct nematodes and humans.** *C. elegans* (Ce), *H. sapiens* (Hs), *B. malayi* (Bm), *T.spiralis* (Ts), *M. incognita* (Mi).
(EPS)

Figure S2 **Number of total kinases within each species/group of compounds.** The curated kinases were considered for the species. Each compound that mapped to an identified kinase target in DrugBank via HMM was considered (cutoff 0.1). The compounds that target a protein in each group were counted.
(EPS)

Figure S3 **Example** ***C. elegans*** **dose response curves for each compound.**
(EPS)

Figure S4 **Example** ***H. contortus*** **dose-response curves for each compound.**
(EPS)

Figure S5 **Alignment, structure, and experimental data for p38 MAP kinase (CMGC _group1).** A. 1DI9 with DB02984 bound. Residues within 10 Å of the active site that differ between mammals and nematodes are colored in grey. B. 1DI9 alignment of Tsp_03128 (*T. spiralis*), Mh10g200708_Contig883_17801_19594 (*M. hapla*), 14956.m00538 (*B. malayi*), B0218.3 (*C. elegans*), CBN12041 (*C. brenneri*), CBG01555 (*C. briggsae*), NP_001095644.1 (*B. taurus*), P70618.3 (*R. norvegicus*), ENSMUSP00000004990 (*M. musculus*), ENSP00000229795 (*H. sapiens*), NP_001003206.1 (*C. familiaris*), 1DI9. C. Expression data from *B. malayi* microarray data [66] for p38 orthologs in *B. malayi*. D. Percent reduction in microfilarial release in *B. malayi* exposed to Compound **12** *in vitro*. E. Percent reduction in viability in *B. malayi* when exposed to Compound **12**.
(EPS)

Figure S6 **Kinases in** ***C. elegans*** **(cutoff 0.1) mapped to the major KEGG pathways.**
(EPS)

Figure S7 **Tree of CK1 alpha and gamma with distance calculated using percent identity.** Compound **11** was found to also inhibit TcCK1.1 in *T. cruzi in vitro* [45], as well as aminoglycoside kinase in *Legionella pneumophila* [46]. A sequence comparison revealed that TcCK1.1 is closer to CK1 alpha than CK1 gamma. At this time, there is no data available for **11** binding CK1 alpha in *H. sapiens*. Given the results with *T. cruzi*, the **11** may be targeting the CK1-alpha-like nematode proteins, C03C10.1 and F46F2.2 as well, which could provide further opportunities for specific targeting.
(EPS)

Figure S8 **Alignment and structure of casein kinase I (CK1_group2).** A. Structure of 2C47 (*H. sapiens* casein kinase I gamma2) with **3** bound (green). The structure of 2CSN (*S. pombe* casein kinase I) was superimposed with 2C47 using Pymol [67]. 2CSN has **11** bound (yellow). The residues that differ between mammals and nematodes are highlighted in grey. Sequence analysis between mammals and nematodes revealed some subtle differences within 10 Å that could be potentially exploited to make a slightly more specific compound. B. Dose-response curve of **3** in *C. elegans*. C. The alignment of prot_Minc02294 (*M. incognita*), Mh10g200708_Contig69_9047_6539 (*M. hapla*), 14971.m02829 (*B. malayi*), CBN30117 (*C. brenneri*), CBG12215 (*C. briggsae*), CRE23960 (*C. remanei*), Y106G6E.6 (*C. elegans*), Tsp_05423 (*T. spiralis*), ENSP00000307753 (*H. sapiens*), ENSMUSP00000082561 (*M. musculus*), EHH54474.1 (*M. fascicularis*), NP_001094526.1 (*B. taurus*), and NP_001029042.1 (*R. norvegicus*).
(EPS)

Figure S9 **Expression of** ***B. malayi*** **orthologs for casein kinase I and results from testing Compound 11 in** ***B. malayi*** **.** A. Expression data from *B. malayi* microarray data [66] for casein kinase I alpha orthologs in *B. malayi*. B. Percent reduction in viability in *B. malayi* male and female worm exposed to Compound **11** *in vitro*. Two *B. malayi* orthologs of CK1 alpha, 14972.m07128 and 14979.m04526, were much more highly expressed in males versus females (log ratio of 0.68 and 0.61, respectively, compared to an average log ratio of −0.13 and 0.15, respectively). However, the *B. malayi* ortholog of CK1 gamma (14971.m02829) was much more highly expressed in female and MF (log ratio of 0.48 and 0.82, respectively compared to an average log ratio of −0.07) [40].
(EPS)

Table S1 **The** ***B. malayi*** **kinases in the minimal pan-phylum containing the 68 orthologous groups.** Multiple *B. malayi* proteins exist in some orthologous groups. The KO, Interpro, and GO ids to which the protein was mapped are also listed.
(XLSX)

Table S2 **The** ***M. incognita*** **kinases in the minimal pan-phylum containing the 68 orthologous groups.** Multiple *M. incognita* proteins exist in some orthologous groups. The KO and GO ids to which the protein was mapped are also listed.
(XLSX)

Table S3 **The** ***T. spiralis*** **kinases in the minimal pan-phylum containing the 68 orthologous groups.** Multiple *T. spiralis* proteins exist in some orthologous groups. The KO and GO ids to which the protein was mapped are also listed.
(XLSX)

Table S4 **The** ***C. elegans*** **kinases in the minimal pan-phylum containing the 68 orthologous groups.** Multiple *C. elegans* proteins exist in some orthologous groups. A description of the protein, the KO and GO ids to which the protein was mapped are also listed. Tissue expression as well as RNAi classification in *C. elegans* are also listed.
(XLSX)

Table S5 **The top orthologous groups from the minimal kinome listed with the respective** ***C. elegans*** **protein, the kinase classification, E-value from the HMM, DrugBank target ID and compounds known to bind to the target, KO and IPR IDs, information regarding tissue expression in** ***C. elegans*** **, RNAi phenotype, and stage expression RNAseq data analyzed within a stage and across different stages.**
(XLSX)

Table S6 **Top orthologous groups, the drug bank compound ids associated with the targets within the orthologous group, drug common name, drug use, treatment the drug is used to treat, and category of the drug.**
(XLSX)

Table S7 **Compounds tested in** ***C. elegans*** **, catalog number used, kinase classification of compounds target from drug bank, the phenotype the compound caused in** ***C. elegans*** **, and the EC50 results in** ***C. elegans*** **listed in ppm concentrations.**
(XLSX)

Video S1 ***C. elegans*** **control.**
(WMV)

Video S2 ***C. elegans*** **exposed to compounds 5.**
(WMV)

Video S3 ***C. elegans*** **exposed to compounds 6.**
(WMV)

Video S4 ***C. elegans*** **exposed to compounds 8.**
(WMV)

Video S5 ***H. contortus*** **control.**
(WMV)

Video S6 ***H. contortus*** **exposed to compound 5.**
(WMV)

Video S7 ***H. contortus*** **exposed to compound 6.**
(WMV)

Video S8 ***H. contortus*** **exposed to compound 8.**
(WMV)
